# Leak Following Zenker’s Peroral Endoscopic Myotomy (Z-POEM): Non-operative Management Strategy of a Large Undrained Submucosal Cavity

**DOI:** 10.7759/cureus.99070

**Published:** 2025-12-12

**Authors:** Toshitaka Hoppo, Phillip R Purnell, Ioannis Kontopidis, Petros C Benias

**Affiliations:** 1 Department of Surgery, Division of General Surgery, Rutgers Robert Wood Johnson Medical School, New Brunswick, USA; 2 Department of Otolaryngology-Head and Neck Surgery, Rutgers Robert Wood Johnson Medical School, New Brunswick, USA; 3 Department of Surgery, Division of Thoracic Surgery, Rutgers Robert Wood Johnson Medical School, New Brunswick, USA; 4 Department of Medicine, Division of Gastroenterology, Rutgers Robert Wood Johnson Medical School, New Brunswick, USA

**Keywords:** cervical esophageal perforation, mucosal dehiscence, undrained submucosal cavity, zenker’s diverticulum, z-poem

## Abstract

Zenker’s peroral endoscopic myotomy (Z-POEM) is a safe and effective endoscopic therapy for Zenker's diverticulum. The most common serious complication was reported to be perforation, which can often be treated non-operatively, although the optimal management strategy has not been established. Here, we present a case of a 72-year-old female patient who had a leak following Z-POEM to treat a 1.5-cm, small Zenker’s diverticulum with concomitant hypopharyngeal stricture. Tension on a mucosal closure caused mucosal dehiscence, which communicated to a deep submucosal tunnel and led to an 8-cm contained undrained cavity posterior to the esophagus as demonstrated by computed tomography. Once it was recognized that the deep cavity was contained and granulating without clinical signs of mediastinitis, endoscopic dissection of the mucosa over the deep, undrained cavity was performed to provide continuous, optimal drainage, thus significantly facilitating the healing process with granulation formation and re-epithelization of the esophageal lumen. A contained leak after Z-POEM without signs of mediastinitis can be treated conservatively. It is important to have short-interval follow-up after Z-POEM to treat small Zenker’s diverticula, as the closure in such cases can fail early due to tension.

## Introduction

Zenker’s diverticulum (ZD) is a rare outpouching of the pharyngeal mucosa through Killian’s triangle in the proximal esophagus. Hypertrophic cricopharyngeal muscle is thought to increase intraluminal pressure, leading to the development of ZD [1]. Therefore, it is crucial to completely divide the cricopharyngeal muscle, which creates a septum between ZD and the esophagus.

Zenker's peroral endoscopic myotomy (Z-POEM) has been widely accepted as a safe and effective endoscopic therapy for the management of ZD [2-6]. However, mucosal closure of Z-POEM is often challenging due to a limited working space in the hypopharynx. The pooled data in a recent meta-analysis demonstrated that the complication rate after Z-POEM was 9.4%, and the most common serious complication was perforation, which occurred in 2.25% [2]. Since the submucosal space is adjacent to the danger space between the alar and prevertebral fascia that extends freely from the skull base all the way to the posterior mediastinum, a leak into the submucosal space could cause life-threatening complications such as mediastinitis [5].

A few cases of successful endoscopic management using a transnasal drainage tube into the submucosal cavity with either a stent placement or a vacuum-assisted closure (VAC) device implant have been reported [7,8]; however, the management strategy of leak following Z-POEM has not been established. Here, we present a case of leak following Z-POEM, which was successfully treated by endoscopic management without stents or VAC devices.

## Case presentation

**Figure 1:**
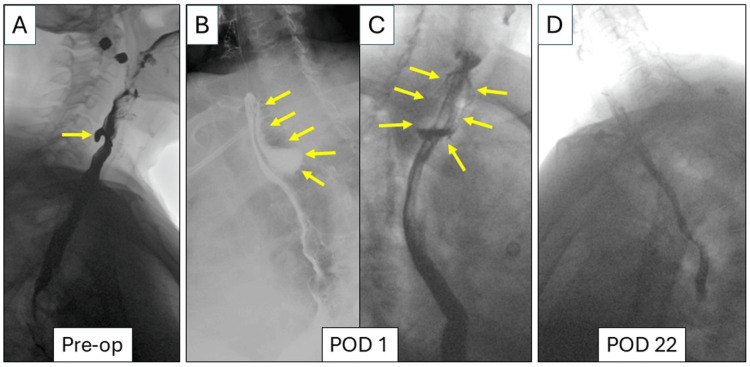
Esophagram findings (A) Preoperative (Pre-op) esophagram showed a 1.5-cm Zenker’s diverticulum (yellow arrow). (B) Lateral view and (C) anterolateral view of esophagram on postoperative day (POD) 1 demonstrated a leak through the mucosal closure with a 4 x 2 cm contained collection posteriorly to the esophagus (yellow arrows). (D) On POD 22, esophagram did not demonstrate any contained leak.

A 72 year-old female patient with a history of total thyroidectomy for Graves’ disease presented with cervical dysphagia and choking sensation requiring the Heimlich maneuver. Esophagram demonstrated a 1.5-cm ZD (Figure 1A), and she was then referred to our tertiary center for Z-POEM.

**Figure 2:**
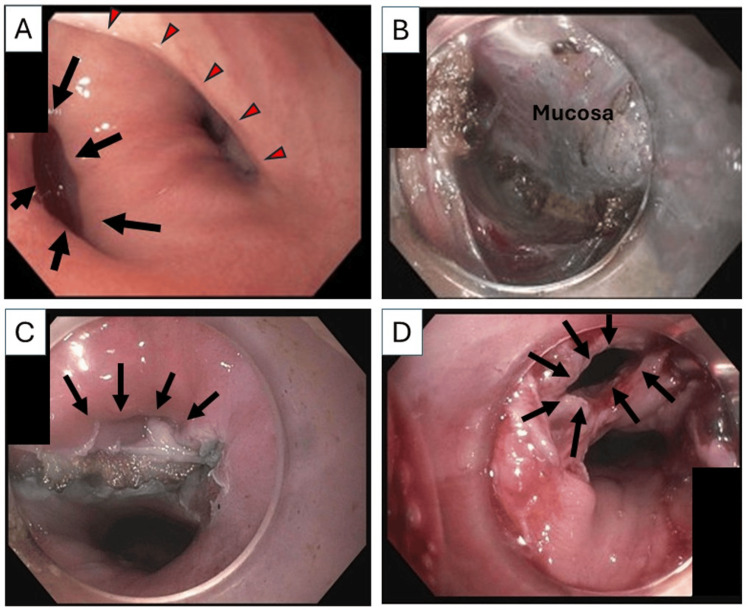
Pre- and post-procedure endoscopic findings (A) Preoperative esophagogastroduodenoscopy showed a 1.5-cm Zenker’s diverticulum (black arrows) and concomitant hypopharyngeal stricture (red arrow heads). (B) Completion of cricopharyngeal and 1-2 cm proximal esophageal myotomy. (C) Following myotomy, the small Zenker’s diverticulum nearly resolved (arrows), with insufficient mucosal tissue available for tension-free closure. (D) On postoperative day 1, a partial dehiscence of the mucosotomy due to inflamed, friable mucosa (arrows) was seen.

At the time of Z-POEM, the patient was found to have a 1.5-cm ZD with concomitant, mild hypopharyngeal stricture (Figure 2A). A 1.5-cm mucosotomy was created horizontally on the septum, and access to the submucosal space was created. A 3-cm submucosal tunneling was performed, and the cricopharyngeal muscle was divided. Subsequently, myotomy was extended 1-2 cm to the proximal esophagus to ensure a complete division of the cricopharyngeal muscle (Figure 2B). Mucosal closure with clips was difficult due to concomitant hypopharyngeal stricture and insufficient mucosal flaps to close without tension (Figure 2C), but closure of mucosotomy using four MANTIS™ clips (Boston Scientific Corporation, Marlborough, Massachusetts, United States) was eventually successfully accomplished. Postoperatively, the patient complained of neck and left anterior chest pain with mild crepitus in the neck, but remained afebrile and hemodynamically stable.

On postoperative day (POD) 1, esophagram demonstrated a leak at the apex of the mucosotomy with an associated 4 x 2 cm contained collection posterior to the esophagus (Figures 1B, 1C). She underwent esophagogastroduodenoscopy (EGD), which revealed a partial dehiscence of the mucosotomy due to severely inflamed, friable mucosa (Figure 2D).

**Figure 3:**
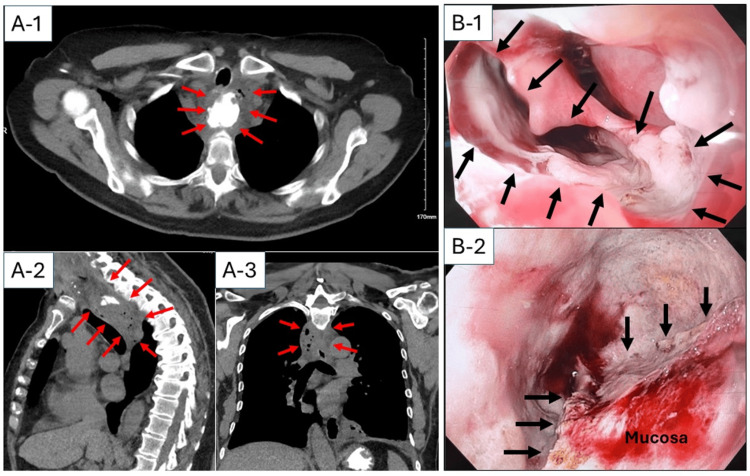
Computed tomography (CT) and endoscopic findings on postoperative day 5 (A-1) axial, (A-2) sagittal, and (A-3) frontal views of CT scan  demonstrated a 4.3 x 3.3 x 7.8 cm contained fluid collection with gas formation posterior to the proximal esophagus (red arrows). (B) EGD revealed a 15 x 10 mm complete mucosal dehiscence (B-1, arrows) with 4 cm deep contained cavity posterior to the esophagus (B-2, arrows indicate the edge of myotomy).

Re-approximation of the tissue, even with endoscopic suturing, was not feasible due to persistent inflammation and a lack of working space. Although leukocytosis (17,000 cells/µL) was seen, the patient remained afebrile and hemodynamically stable without worsening neck or chest pain. Therefore, non-operative management with nil per os (NPO), antibiotics, and total parenteral nutrition (TPN) was initiated. On POD 5, computed tomography (CT) demonstrated a 4.3 x 3.3 x 7.8 cm contained fluid collection with gas formation posterior to the proximal esophagus (Figure 3A-1, 3A-2, 3A-3). Subsequently, she was brought to the operating room for possible open drainage. EGD at that time showed a 15 x 10 mm complete mucosal dehiscence (Figure 3B-1) with a 4 cm deep contained cavity posterior to the esophagus (Figure 3B-2), where abundant purulent fluid and debris were observed. The cavity appeared contained on intraoperative fluoroscopy with early signs of granulation. Neck exploration was therefore deferred, and a nasogastric tube with side holes was placed next to the mucosal dehiscence, with the expectation to drain the contained cavity. Since she was hemodynamically stable, non-operative management was continued.

**Figure 4:**
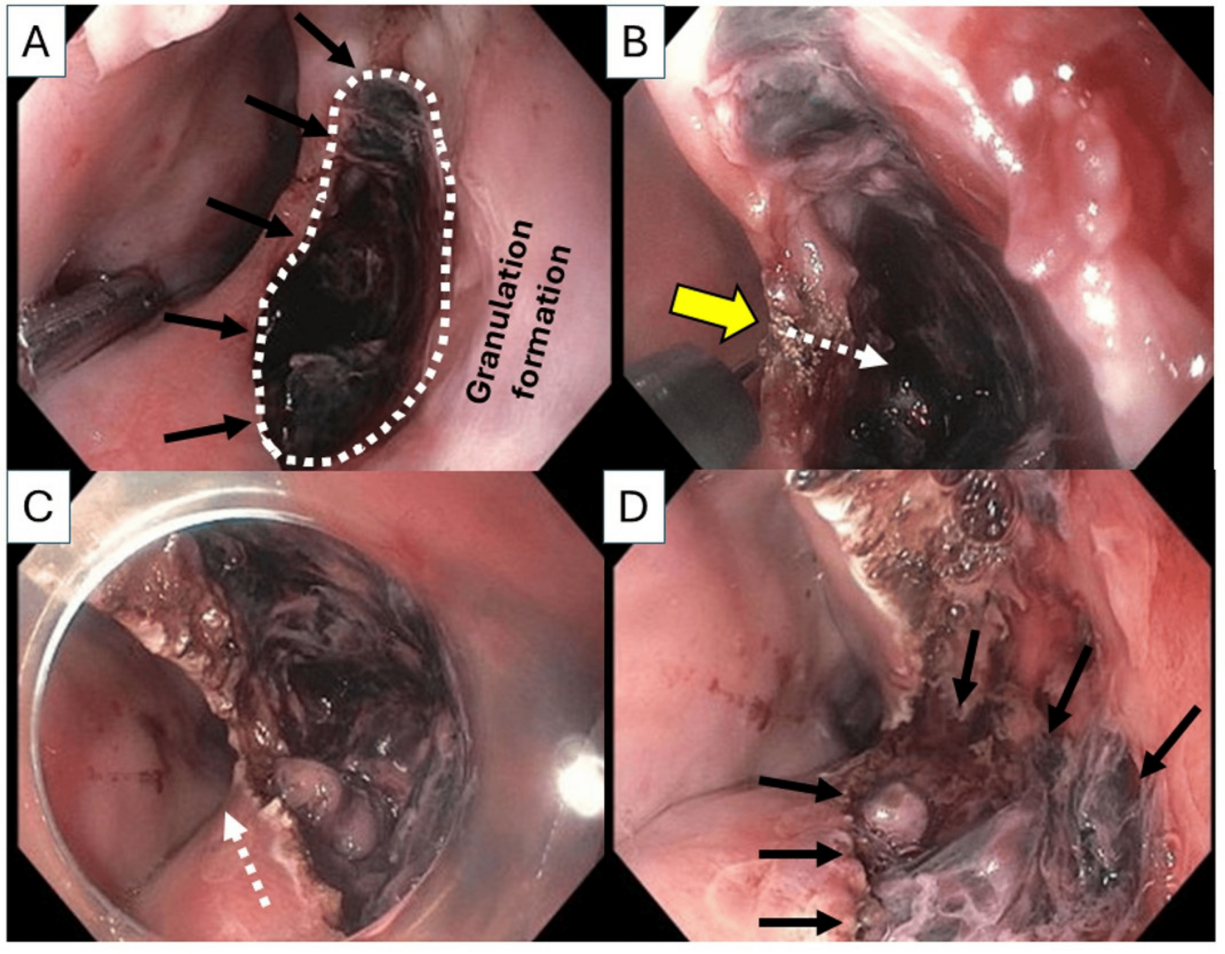
Longitudinal mucosal dissection over the deep undrained cavity on postoperative day 13 (A) The mucosal defect (arrows) was extended distally (15 x 30 mm) with granulation formation behind the mucosal defect and a persistent and deep cavity (circled by a dotted white line). (B) Initiation of mucosal dissection (yellow arrow) over the deep undrained cavity using a Triangle-Tip Knife. A dotted white arrow indicates the direction of mucosal dissection towards the bottom of deep cavity. (C) Longitudinal mucosal dissection was performed distally (a dotted white arrow indicates the direction of mucosal dissection). (D) At the completion of 3-cm longitudinal mucosal dissection to the bottom of undrained cavity, the entire undrained cavity was fully opened to the esophageal lumen (arrows indicate purulent fluid and debris that were in the undrained cavity).

On POD 13, repeat EGD revealed that the mucosal defect was extended distally (15 x 30 mm) with continued granulation, and a persistent and deep cavity (Figure 4A). A decision was therefore made to widely open the undrained deep cavity into the esophageal lumen. This required a 3-cm longitudinal mucosal dissection over the undrained cavity using a Triangle-tip knife (Figure 4B, 4C). At the completion of a 3-cm longitudinal mucosal dissection to the bottom of the undrained cavity, the entire undrained cavity was fully opened to the esophageal lumen (Figure 4D). A 20-Fr percutaneous gastrostomy tube was also placed for enteral nutrition.

**Figure 5:**
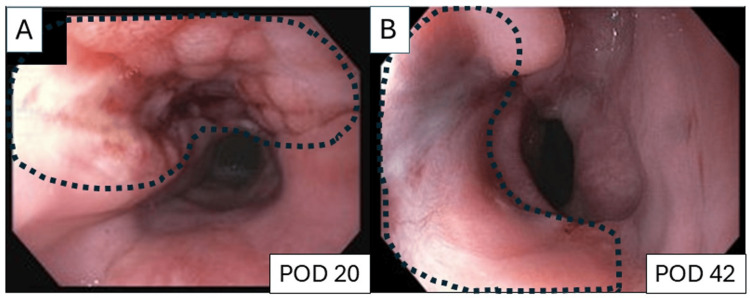
Follow-up endoscopic findings (A) On postoperative day (POD) 20, EGD revealed a 15 x 40 mm mucosal defect with granulation formation without any undrained cavity (circled by a dotted line). (B) On POD 42, the mucosal defect was completely covered with healthy esophageal epithelium (circled by a dotted line).

On POD 20, EGD revealed a large (15 x 40 mm) mucosal defect with granulation formation without an undrained cavity (Figure 5A). Then, the nasogastric tube was removed, and a clear liquid diet was initiated. On POD 22, the esophagram was negative for any leak (Figure 1D), and the patient was discharged home on a full liquid diet and tube feeding via gastrostomy tube. On POD 42, EGD demonstrated complete closure of the mucosal defect with healthy esophageal epithelium (Figure 5B). No ZD was observed. Subsequently, diet was advanced as tolerated. At the follow-up on POD 58, cervical dysphagia and choking sensation had resolved, and she tolerated a regular diet. The gastrostomy tube was therefore removed.

## Discussion

The cervical esophagus is bordered by the trachea anteriorly, carotid sheaths laterally, and a series of fascial layers posteriorly. These layers create potential spaces for infectious spread. The danger space, situated between the alar and prevertebral fascia, is the most critical pathway because it extends freely from the skull base all the way to the posterior mediastinum, without barriers. While lateral spread may be limited by esophageal attachments to prevertebral fascia, vertical communication through the danger space poses the greatest risk for mediastinitis and empyema [9]. On the other hand, these fascial planes could help contain contamination and reduce the risk of systemic sepsis [10,11], despite the septic contents and continuous movement with swallowing and breathing in the hypopharyngeal area [12]. Therefore, a lower mortality rate of cervical esophageal perforation has been reported when compared to intrathoracic or intra-abdominal esophageal perforations [5].

Cervical esophageal perforation can often be managed non-operatively [2,3,5,13]; however, there are a few reports describing the management of a perforation following a Z-POEM. After Z-POEM, mucosal dehiscence can easily communicate to a deep submucosal space where there is no esophageal wall due to myotomy. Therefore, it is crucial to achieve optimal drainage from a deep submucosal space for successful endoscopic management. In previously published case reports, a transnasal placement of a drainage tube into the cavity with either stent placement or a polyurethane sponge with a VAC device implant was performed, and was successful [7,8]. In our case, a longitudinal dissection of the mucosa over the deep undrained cavity provided optimal drainage and significantly facilitated healthy granulation formation and re-epithelization of the esophageal lumen, and neither a stent nor a VAC device was required. Previously, a case of a 6 x 2 cm mucosal sloughing over the submucosal tunnel without an undrained cavity in a patient with achalasia was reported, and this large mucosal defect self-closed without a stent [14]. This suggests that the key to successful management in this setting would be to effectively eliminate an undrained cavity so the healing process can be facilitated without the need for stents or VAC devices. Therefore, it is beneficial to fully open the cavity to achieve continuous, optimal drainage into the esophageal lumen as soon as it is recognized that the cavity is contained and granulating. Stents are typically a difficult salvage technique for these types of perforations because of the location in the cervical esophagus [15]. In addition, closure of the Z-POEM has typically been more difficult due to the lack of space as compared to other POEM techniques. Therefore, minimally invasive salvage procedures need to be explored in these scenarios and should be available at centers performing Z-POEM.

Several single- and multi-center studies have reported the rate of leak after Z-POEM was up to 6%, and most cases were successfully treated non-operatively without any mortality [3,4,6,16]. Post-procedural leaks tend to occur during the initial learning curve, and potential risk factors have not been recognized. In a multicenter study involving 383 patients who had undergone Z-POEM, Fayyaz and colleagues reported that 10 patients (2.6%) had post-procedural leak, and those with leaks had an average ZD size and myotomy length of 40 ± 11 mm and 49 ± 11 mm, respectively [17]. Case-matched analysis suggested that patients with leaks were more likely to have submucosal fibrosis (30% vs. 0%, p=0.03) and longer myotomy lengths (49 vs. 37 mm, p=0.03) compared to those without leaks, although there was no significant difference in ZD size. On the other hand, Klinger and colleagues reported a case of a patient with a small ZD with a cricopharyngeal bar, who had severe pharyngeal perforation requiring emergent open surgical intervention [18]. Similar to our case, Z-POEM for small ZD may have a higher risk of perforation due to the greatest difficulty of closure; however, small ZD would most benefit from Z-POEM to endoscopically ensure complete division of the cricopharyngeal muscle. Therefore, it is critical to have timely access to post-procedure care, as the failure of closures in such cases can be seen early due to tension. Furthermore, it is important to recognize concomitant pathology such as cricopharyngeal stricture, which can make mucosal closure even more difficult and can be effectively managed with endoscopic dilation alone [19,20].

## Conclusions

In the present case, a small ZD with hypopharyngeal stricture made a mucosal closure difficult, leading to mucosal dehiscence with a deep contained undrained cavity posterior to the esophagus. Although this is a single case report, the key for successful non-operative management of leak after Z-POEM would be to fully open the undrained cavity into the esophageal lumen as soon as it is recognized that the cavity is contained and granulating, unless there is evidence of mediastinitis.
